# Supplementary material for: Digoxin Derivatives Sensitize a Saccharomyces cerevisiae Mutant Strain to Fluconazole by Inhibiting Pdr5p
Source: J Fungi (Basel). 2022 Jul 25;8(8):769. doi: 10.3390/jof8080769 (PMC9330353; doi:10.3390/jof8080769)
Supplement: Supplementary file 1 [file jof-08-00769-s001.zip › jof-1815425-supplementary.pdf]

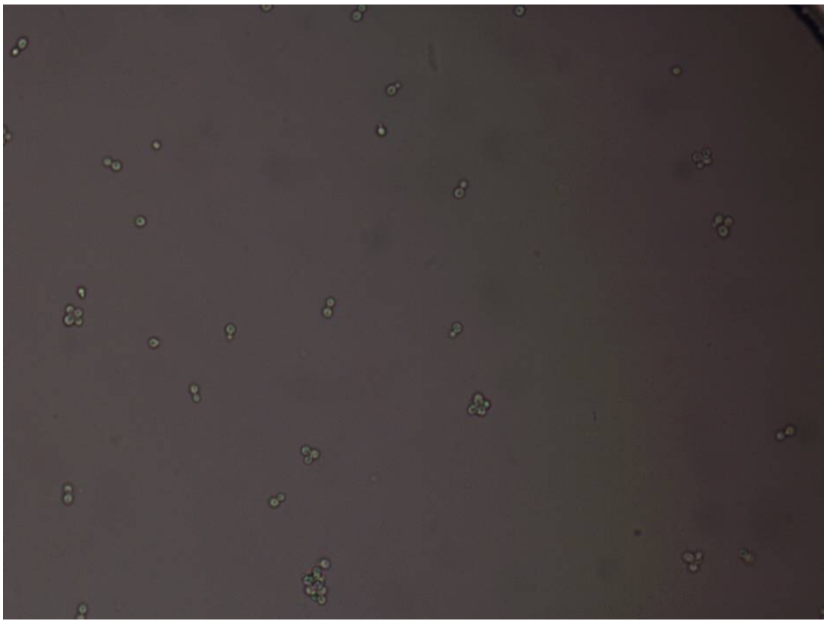

S1. AD/124567 without treatment and with glucose (bright field).

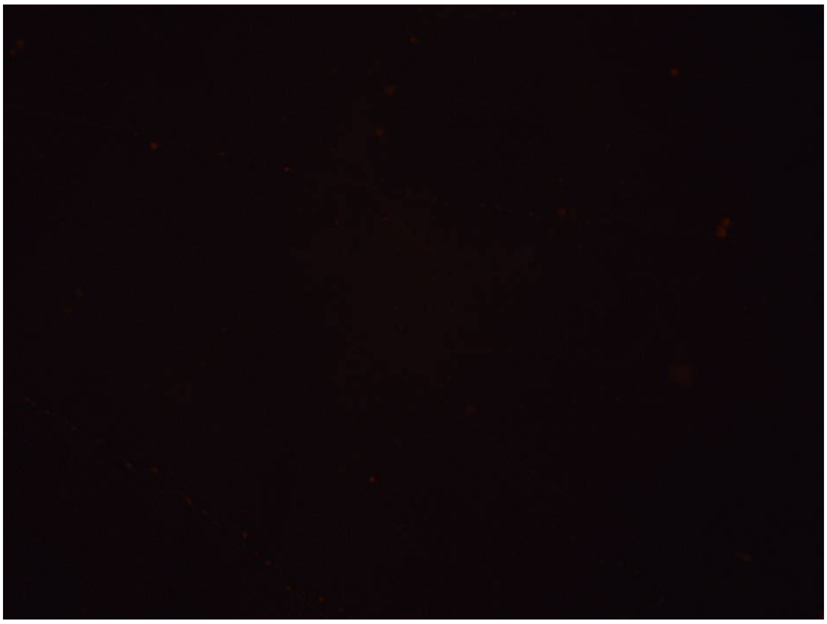

S2. AD/124567 without treatment and with glucose (fluorescence).

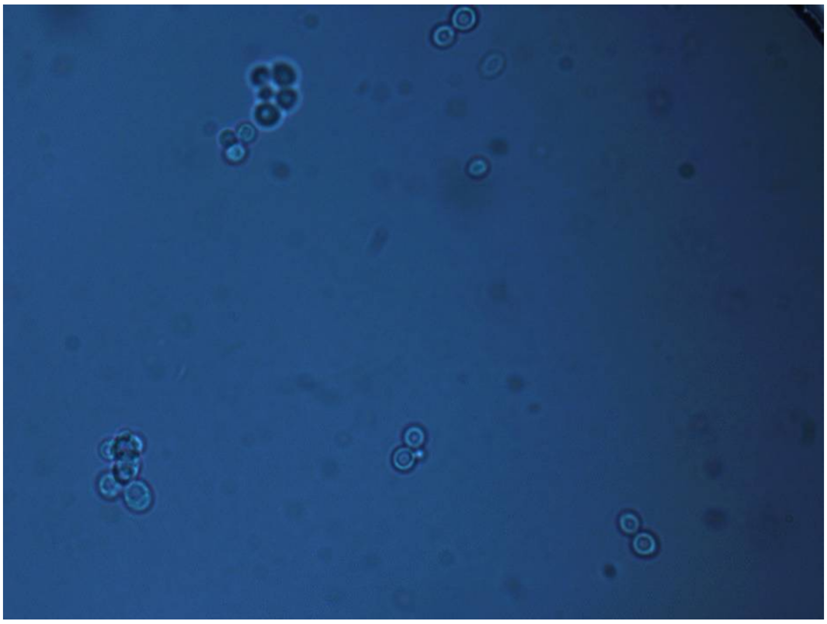

S3. AD/124567 without treatment and without glucose (bright field).

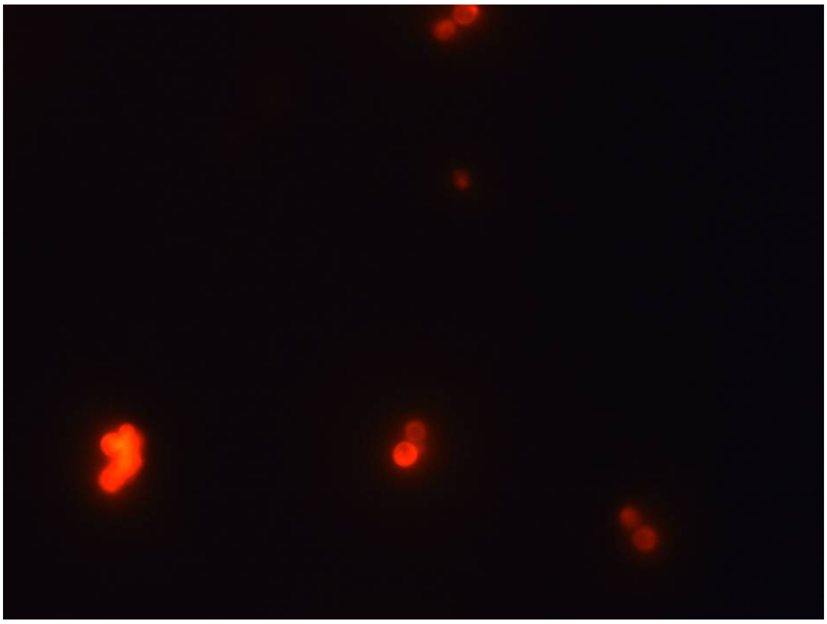

S4. AD/124567 without treatment and without glucose (fluorescence).

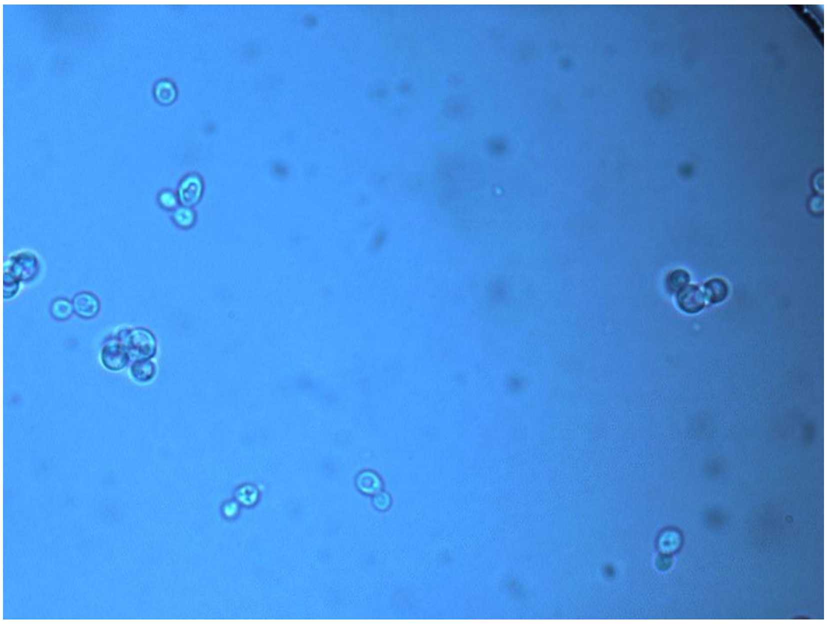

S5. AD/1234567 without treatment and with glucose (bright field).

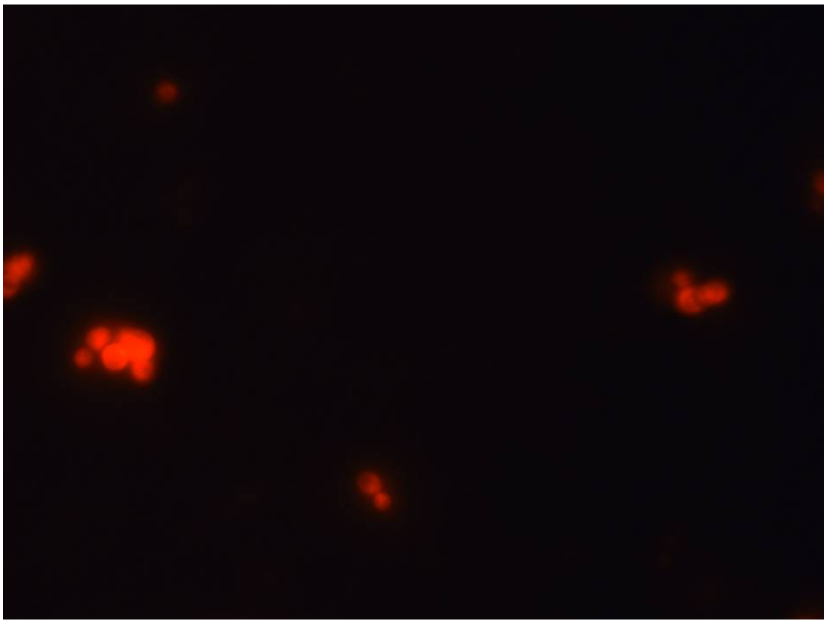

S6. AD/1234567 without treatment and with glucose (fluorescence).

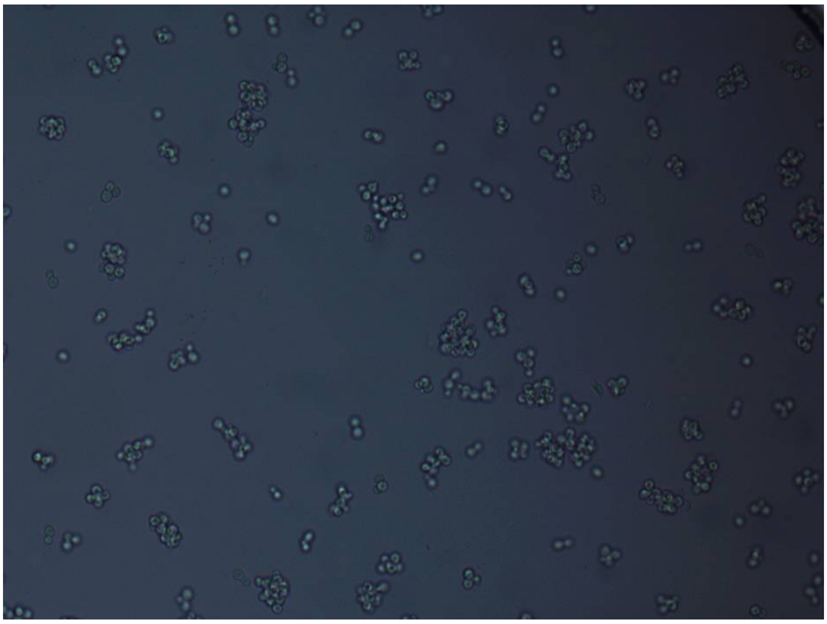

S7. AD/124567 + DGB1 (bright field).

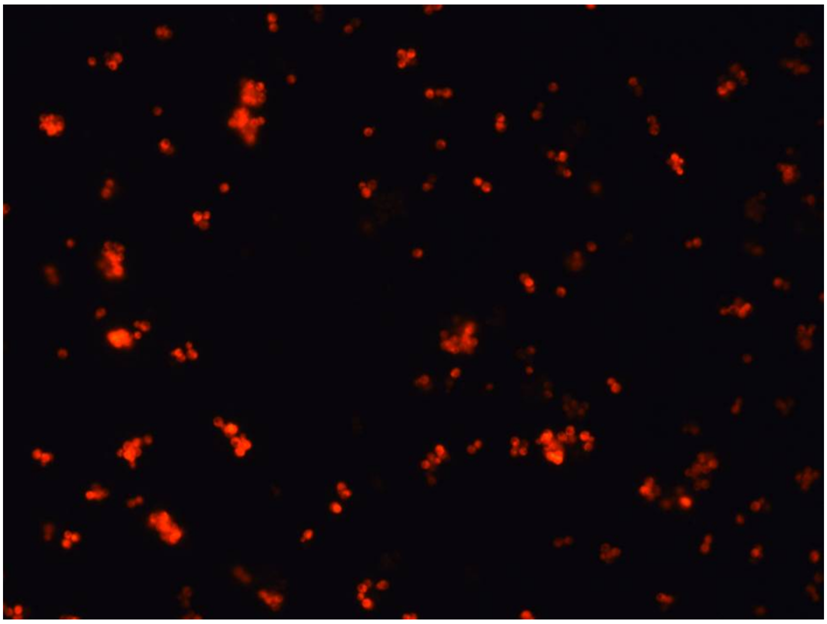

S8. AD/124567 + DGB1 (fluorescence).

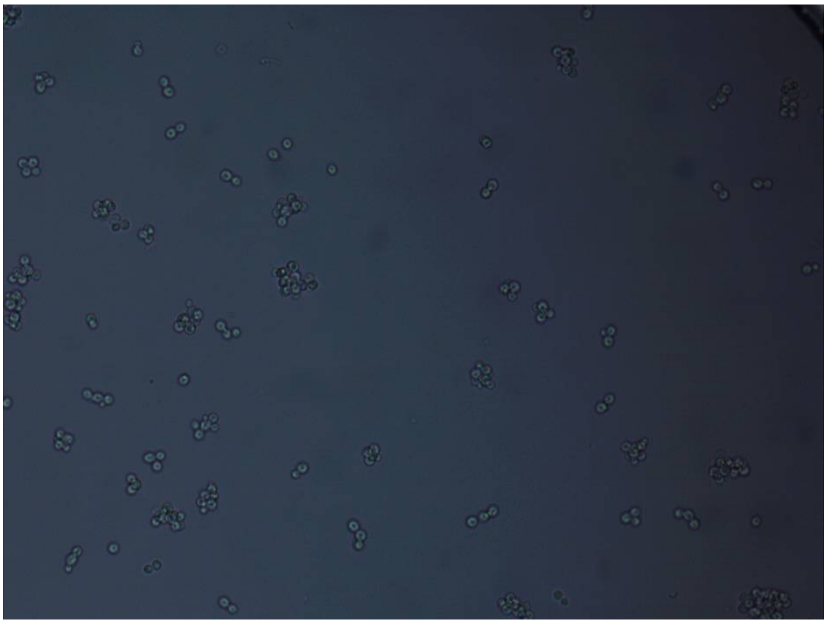

S9. AD/124567 + DGB2 (bright field).

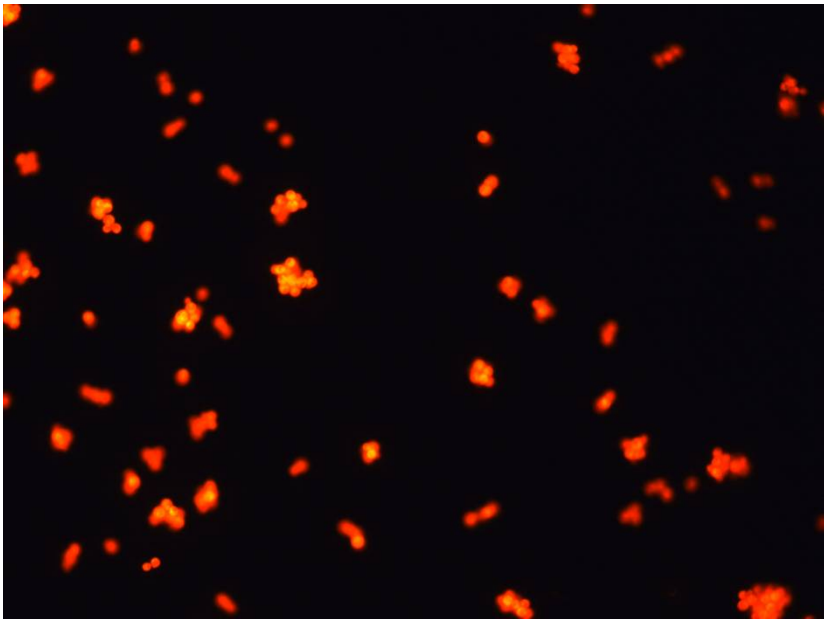

S10. AD/124567 + DGB2 (fluorescence).

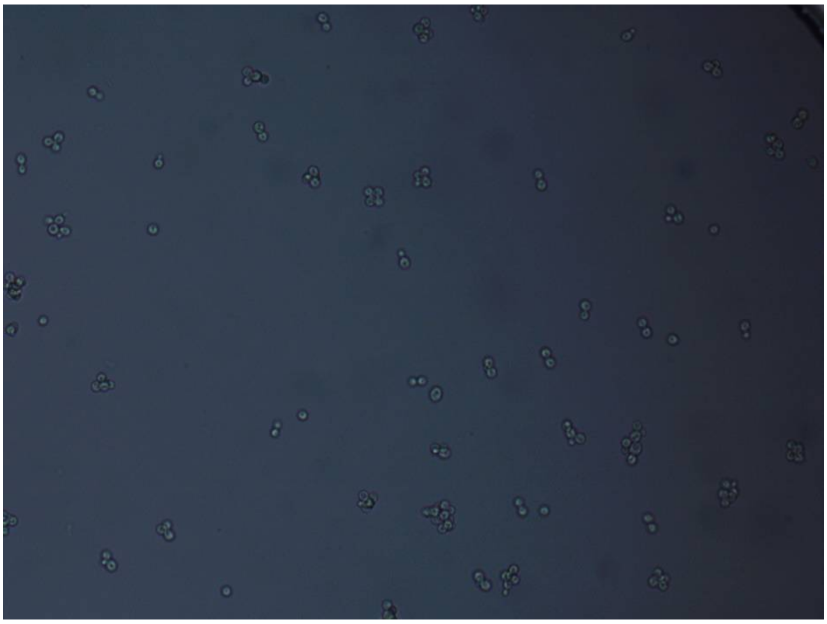

S11. AD/124567 + DGB3 (bright field).

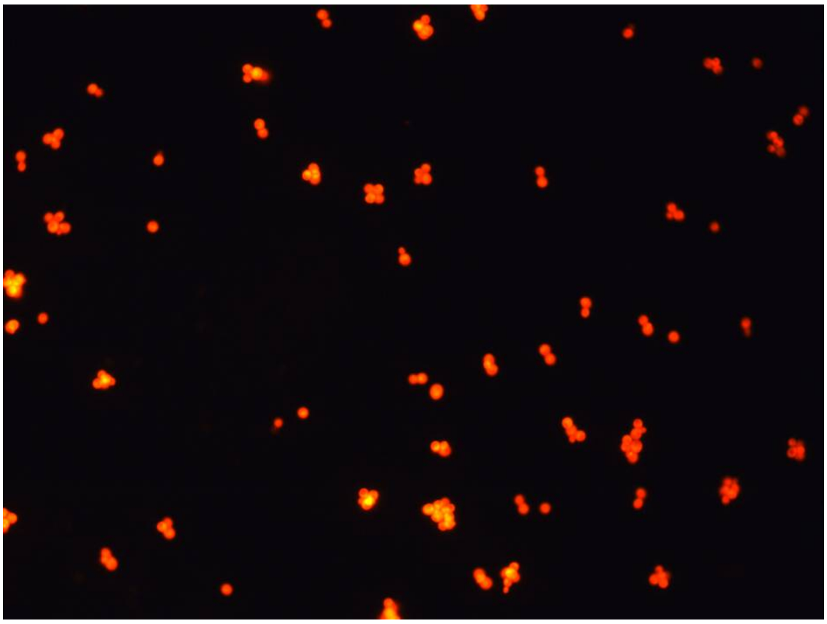

S12. AD/124567 + DGB3 (fluorescence).

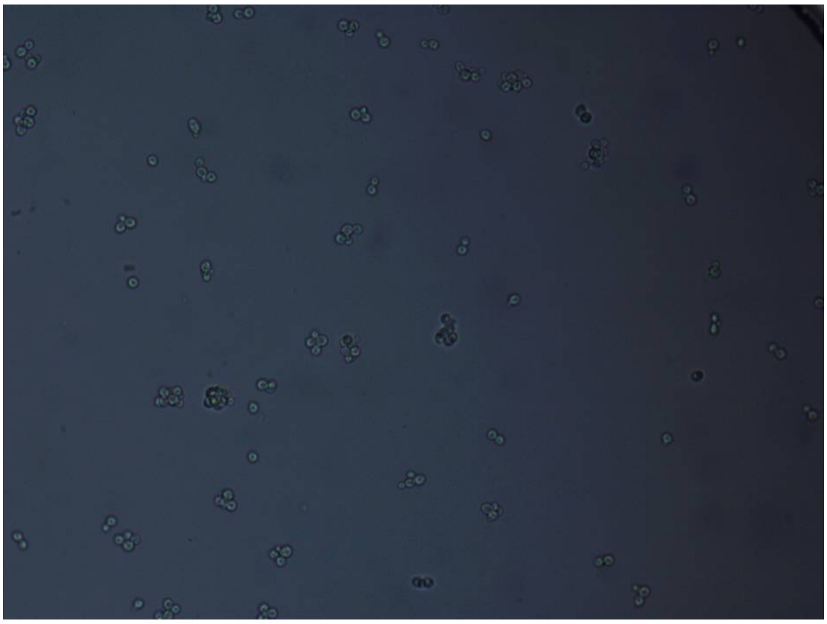

S13. AD/124567 + DGB4 (bright field).

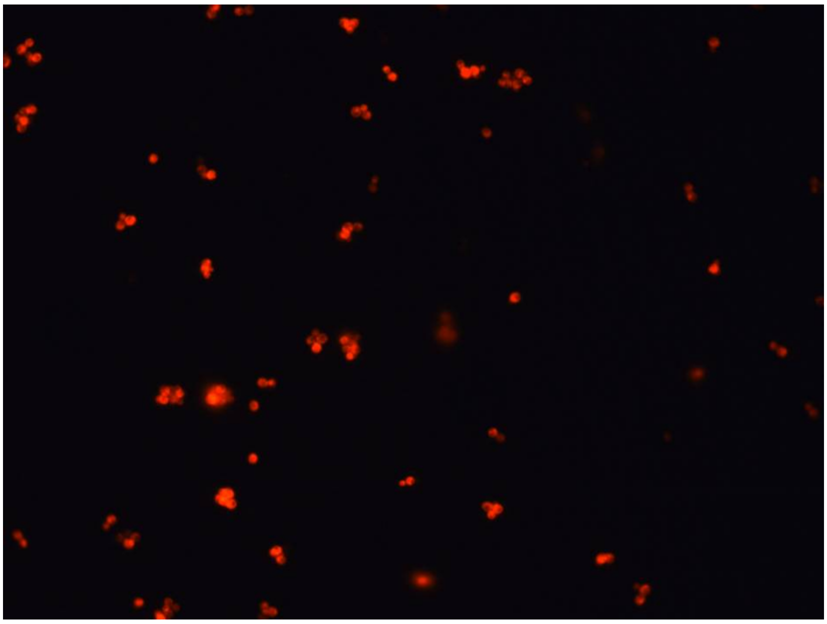

S14. AD/124567 + DGB4 (fluorescence).

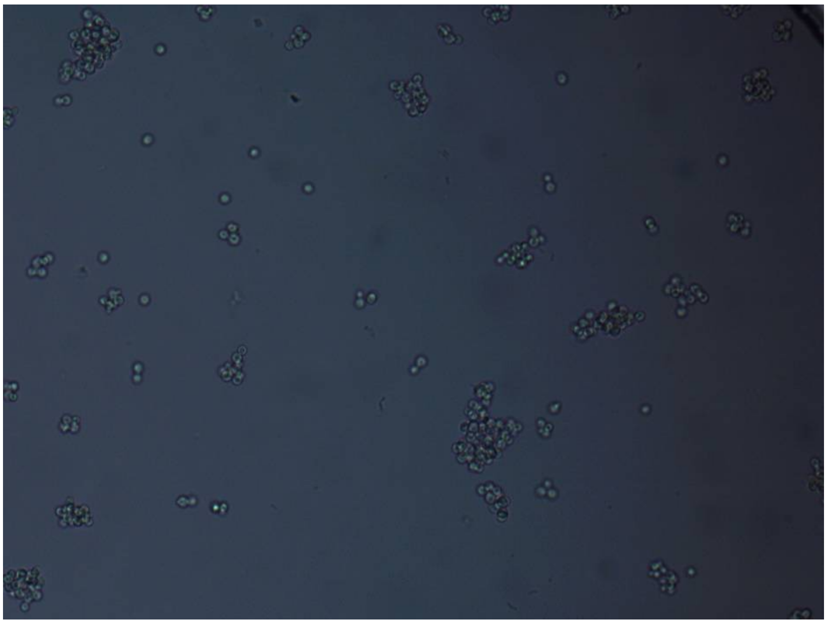

S15. AD/124567 + DGB5 (bright field).

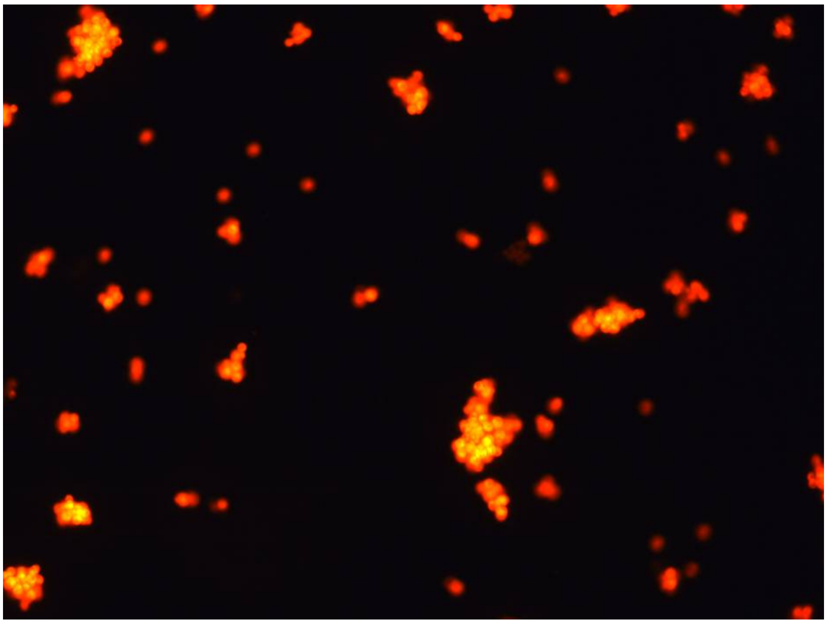

S16. AD/124567 + DGB5 (fluorescence).

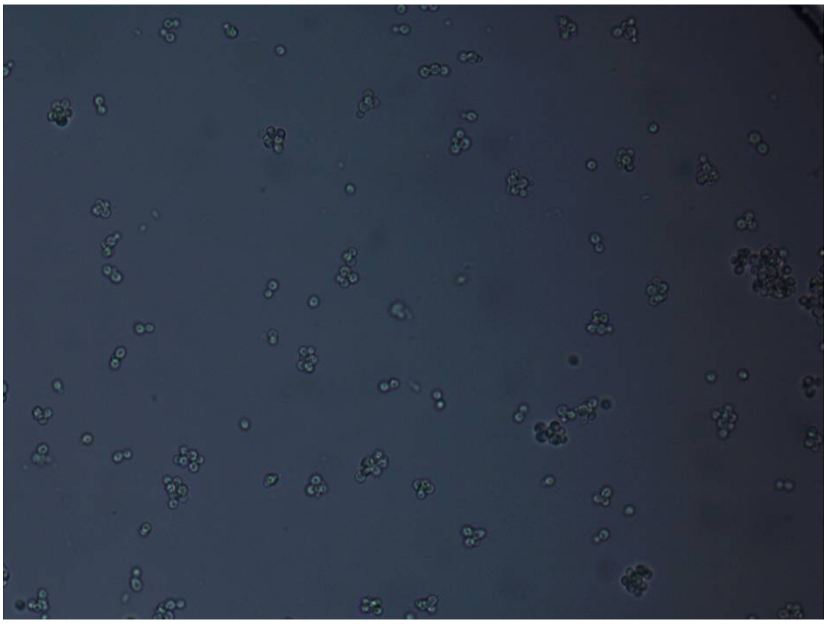

S17. AD/124567 + DGB6 (bright field).

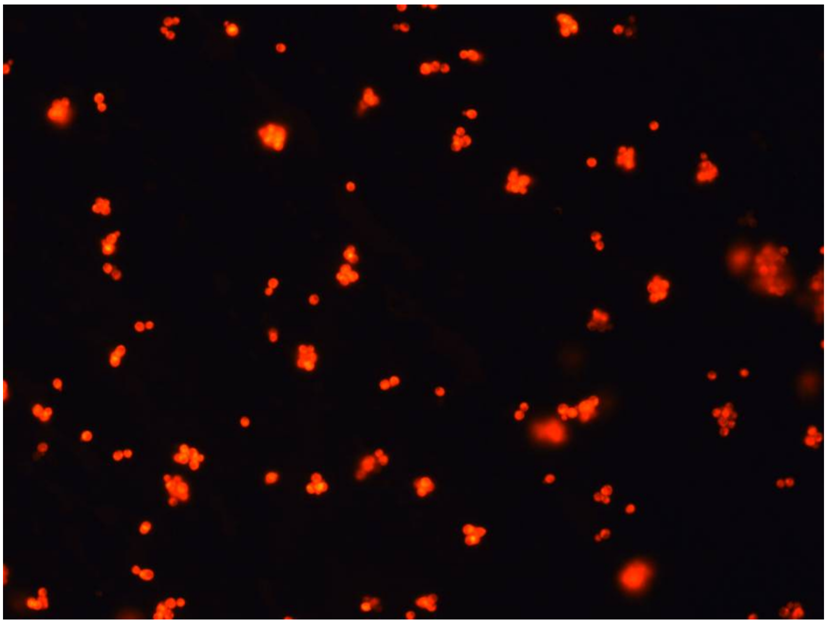

S18. AD/124567 + DGB6 (fluorescence).

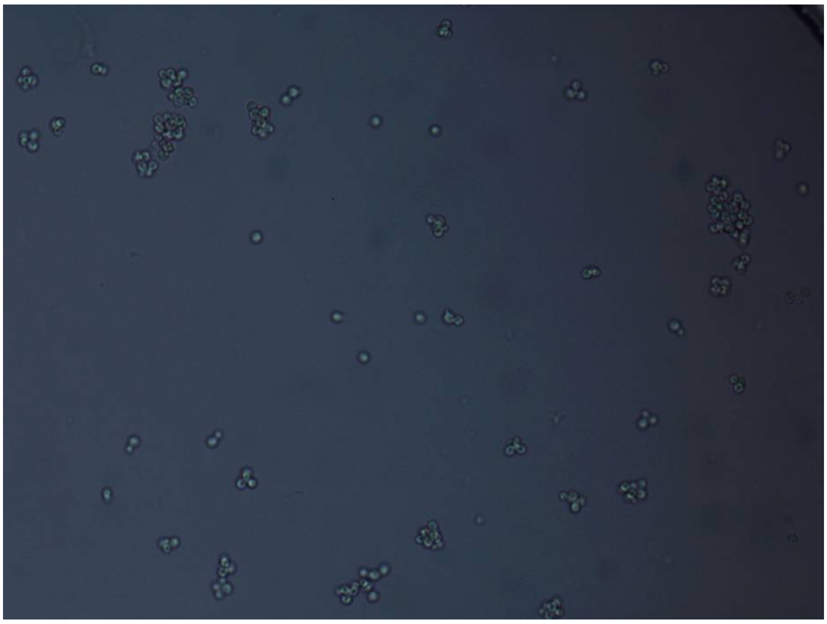

S19. AD/124567 + DGB7 (bright field).

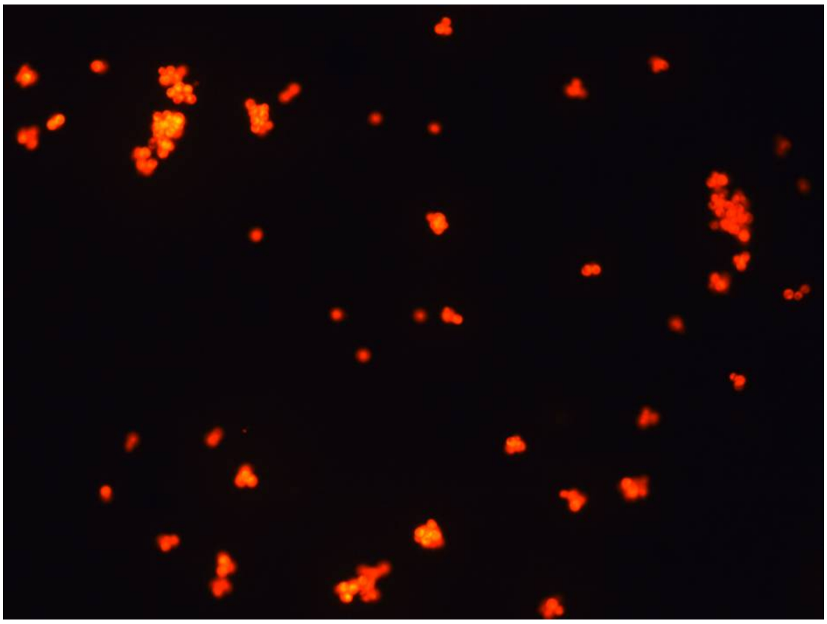

S20. AD/124567 + DGB7 (fluorescence).

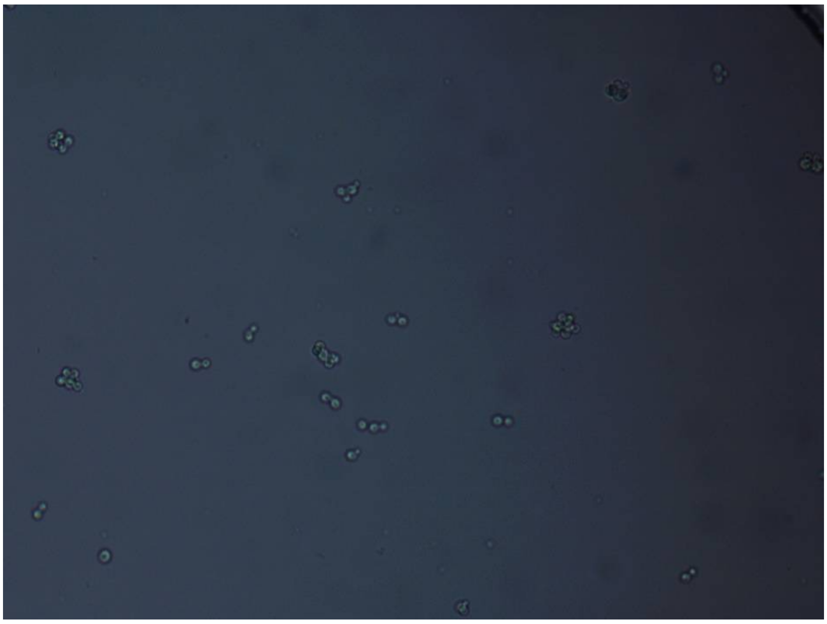

S21. AD/124567 + digoxin (bright field).

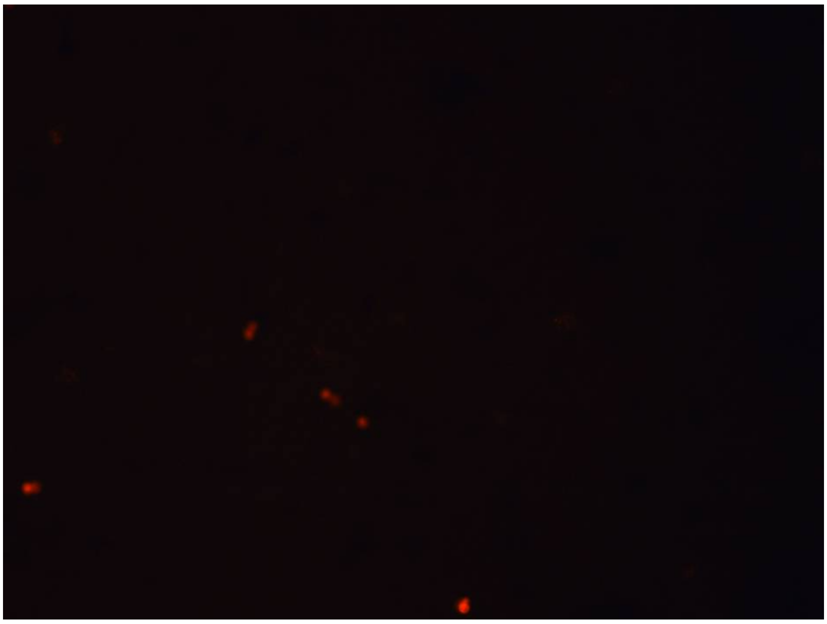

S22. AD/124567 + digoxin (fluorescence).
